# A high-density SNP linkage map reveals a major LG3 QTL hotspot controlling early-maturity–related traits in ridge gourd (*Luffa acutangula*)

**DOI:** 10.3389/fpls.2026.1832490

**Published:** 2026-05-28

**Authors:** Qin Chen, Yuanyuan Guo, Yang Li, Li Zhang, Huanzhong Song, Zheng Zeng, Guijun Su, Jielin Deng, Xiaoyan Sun, Xingxing He, Dexian Kang, Zhendong Chen

**Affiliations:** Guangxi Academy of Agricultural Sciences, Nanning, Guangxi, China

**Keywords:** early maturity, F2 population, high-density genetic map, Luffa, whole-genome resequencing

## Abstract

**Introduction:**

Early maturity is an important breeding target in ridge gourd (*Luffa acutangula*), but its genetic basis remains poorly understood.

**Methods:**

An F_2_ population of 147 plants was developed from a cross between two parental lines contrasting in early maturity and evaluated for six related traits. Whole-genome resequencing of the parents and 144 retained F₂ individuals was used for SNP discovery, binning, high-density linkage-map construction, and QTL analysis.

**Results:**

A high-density genetic linkage map containing 4,035 representative bin markers across 13 linkage groups was constructed. Eight significant QTLs were identified, most of which clustered on LG3 and formed a major multi-trait hotspot. The hotspot was anchored to a 7.03 Mb interval on chromosome 3 containing 136 annotated genes. *Lac03g1231* and *Lac03g1281* were prioritized as biologically plausible candidates.

**Discussion:**

These results provide a genetic framework for dissecting early maturity in ridge gourd and support future fine mapping, developmental-stage expression analysis, functional validation, and marker-assisted breeding.

## 1 Introduction

Ridge gourd (*Luffa acutangula (L.) Roxb.*) is an economically important cucurbit cultivated widely in Asia for vegetable, medicinal, and fiber uses. In commercial production, early maturity is a valuable breeding target because it directly affects cropping efficiency, market supply timing, and adaptation to local growing seasons. In ridge gourd, early maturity is a composite agronomic phenotype involving the timing of flowering initiation, the node position of the first female flower and fruit set, and the interval from sowing to first harvest. These traits are closely related to one another and together determine the practical earliness of a cultivar under field conditions (Wu et al., 2020; Zhang et al., 2020). However, because flowering onset, female flower initiation, and early fruit setting are strongly influenced by photoperiod, temperature, and endogenous hormones, phenotype-based selection alone is often inefficient in breeding programs, especially when genotype-by-environment interactions are substantial (García et al., 2020; Li et al., 2021).

In horticultural crops, QTL mapping has become an effective approach for dissecting complex developmental and reproductive traits controlled by multiple loci. In biparental populations, dense genetic markers combined with robust statistical models enable the identification of genomic regions associated with flowering time, node-related reproductive transitions, sex expression, and harvest-related traits. In cucurbit crops such as cucumber and melon, QTL analyses have revealed that earliness-related traits often show a multi-locus architecture, while some genomic regions exhibit relatively large effects and may influence multiple correlated traits simultaneously. Such multi-trait QTL clusters are of particular interest because they may reflect pleiotropy or tight linkage among genes involved in coordinated developmental transitions. Therefore, high-density linkage mapping provides an important framework for resolving the genetic basis of early maturity and for developing marker-assisted breeding tools in cucurbits.

Compared with cucumber and melon, the genetic basis of early maturity in ridge gourd remains much less understood. Recent advances in genomic resources for Luffa have substantially improved the feasibility of high-resolution genetic analysis. Chromosome-scale genome assemblies in Luffa now provide a foundation for anchoring linkage groups to physical chromosomes, converting genetic QTL intervals into defined genomic regions, and extracting positional candidate genes for downstream analysis (Wu et al., 2020; Zhang et al., 2020; Pootakham et al., 2021). In parallel, linkage mapping and QTL analyses in Luffa and related cucurbits have demonstrated that complex agronomic traits are genetically tractable when dense marker systems are applied (Liu et al., 2022; Rao et al., 2021), and recent chromosome-level cucurbit genome studies coupled with population-genomic mapping have further expanded the comparative framework for dissecting sex-related and reproductive traits (Wang et al., 2024). Nevertheless, for early-maturity–related traits in ridge gourd—including flowering time, first-flower node position, fruit set, and first harvest timing—major-effect loci and multi-trait hotspot regions have not yet been well characterized.

QTL mapping in biparental populations provides a direct route to connect phenotypic variation with genomic position. Among currently used methods, inclusive composite interval mapping (ICIM) improves detection power and background control and has been widely adopted for identifying main-effect QTL and estimating support intervals in biparental populations (Meng et al., 2015; Wang, 2007). For traits such as early maturity, the value of QTL mapping lies not only in detecting significant loci, but also in anchoring those loci to a reference genome so that physical intervals can be defined, candidate genes can be prioritized, and linked markers can be developed for future breeding applications. This is especially important when an initial QTL interval spans several megabases and contains many annotated genes, making additional prioritization necessary before fine mapping and functional validation.

Biologically, several conserved regulatory pathways provide plausible mechanistic explanations for QTL affecting early-maturity–related traits in cucurbits. In angiosperms, floral transition is coordinated by regulatory modules integrating endogenous and environmental signals. The florigen FLOWERING LOCUS T (*FT*) and related PEBP family proteins are central integrators of flowering; *FT* protein produced in leaves moves to the shoot apical meristem and promotes floral initiation through interaction with *FD* and downstream transcriptional programs (Abe et al., 2005; Corbesier et al., 2007; Kardailsky et al., 1999; Kobayashi et al., 1999). Further studies have shown that *FT* mobility and long-distance transport are dynamically regulated, linking systemic signaling with developmental timing (Endo et al., 2018). In vine crops, these flowering regulators may also affect node-based reproductive transitions along the main stem.

In Cucurbitaceae, reproductive development is additionally shaped by sex-determination pathways, particularly ethylene-related regulation. Ethylene biosynthesis is controlled by ACC synthase (ACS) and ACC oxidase (ACO), and genetic changes affecting these enzymes can alter sex expression and effective femaleness (Boualem et al., 2008, 2015; Chen et al., 2016). In cucumber and melon, ACS family genes such as *ACS11* play key roles in unisexual flower development and can exert large effects on female flower initiation and sex phenotypes (Boualem et al., 2015; Wang et al., 2023). In cucumber, the female locus is associated with ACS-mediated ethylene production, and duplication or recombination events have contributed to female-specific ACS regulation (Knopf and Trebitsh, 2006). Structural variation can also underlie discrete reproductive phenotypes; for example, genome-wide structural-variation mapping identified a copy-number variant associated with reproductive morphology in cucumber (Zhang et al., 2015). Together, these findings indicate that major loci in ethylene biosynthesis and signaling pathways can have interpretable effects on reproductive transitions in cucurbits.

Beyond ethylene-related pathways, meristem identity and determinacy regulators are also relevant to flowering architecture and node patterning. In cucumber, CsTFL1 acts as an important inhibitor of determinate growth and terminal flower formation and competes with CsFT for interaction partners, thereby linking determinacy control with flowering outputs (Wen et al., 2019). More recently, natural and induced alleles disrupting CsTFL1 function have been shown to underlie determinate phenotypes in cucumber, further supporting the role of determinacy regulators in reproductive timing and node distribution (Biernacik et al., 2024). These mechanistic studies suggest that QTL underlying earliness-related traits in ridge gourd may plausibly involve genes functioning in FT/PEBP pathways, determinacy regulation, and/or ethylene-linked reproductive development. However, for ridge gourd, the extent to which major QTL hotspots correspond to any of these pathways remains unknown.

Accordingly, this study quantified phenotypic variation and correlations among six early-maturity–related traits in ridge gourd, constructed a high-density SNP linkage map from whole-genome resequencing, identified QTL and multi-trait hotspot regions associated with early maturity, and anchored the principal hotspot to a physical genomic interval for candidate-gene prioritization. This framework supports subsequent fine mapping, functional analysis, and marker-assisted breeding of early-maturing cultivars.

## 2 Materials and methods

### 2.1 Plant materials and population development

Two ridge gourd (*Luffa acutangula*) parental lines with contrasting early-maturity phenotypes were used in this study: P1-4 (early-maturing) and P2-9 (late-maturing). An F2 mapping population was developed by selfing F_1_ plants derived from the cross P1-4 × P2-9. A total of 147 F2 plants were phenotyped for earliness-related traits. Among them, 144 F2 individuals with high-quality genotype data were retained for genetic linkage mapping and QTL analysis. For resequencing and genotype-quality assessment, 146 samples, including the two parents and the 144 retained F2 individuals, were analyzed.

All plants were grown under normal field management conditions at the experimental site in Nanning, Guangxi, China. Phenotyping was conducted in a single field trial at one location. Young and healthy leaf tissues were collected from each individual at the seedling stage, immediately frozen in liquid nitrogen, and stored at −80 °C until DNA extraction.

### 2.2 Phenotyping of early-maturity–related traits

Six early-maturity–related traits were evaluated in the phenotyped F2 population of 147 plants: first male flower node (FMFN), male flowering time (MFT), first female flower node (FFFN), female flowering time (FFT), first fruit-setting node (FFSN), and first harvest time (FHT).

MFT and FFT were defined as the number of days from sowing to the first appearance of a male flower and a female flower, respectively, on the main vine. FMFN, FFFN, and FFSN were defined as the node positions on the main vine at which the first male flower, first female flower, and first successfully set fruit were observed, respectively. Nodes were counted consecutively from the cotyledonary node upward using a unified scoring rule for all plants.

FHT was defined as the number of days from sowing to the first marketable fruit. A fruit was recorded as harvestable when it had reached normal commercial length and thickness for fresh consumption, retained the tender green marketable stage, and showed no obvious fiber hardening or overmaturity.

Trait observations were conducted continuously during the reproductive transition period to ensure accurate identification of first flowering, first fruit set, and first harvest events. For each trait, descriptive statistics, frequency distributions, and pairwise correlations among traits were calculated to characterize phenotypic variation and their relationships within the F2 population. The two parental lines were phenotyped together with the F2 population using the same scoring criteria. Trait-specific sample sizes varied slightly among phenotypes because a small number of F2 plants lacked scorable records for some first-event traits.

### 2.3 DNA extraction, whole-genome resequencing, read alignment, and SNP calling

Genomic DNA was extracted from frozen leaf tissues using a modified cetyltrimethylammonium bromide (CTAB) method. DNA integrity was evaluated by 1.0% agarose gel electrophoresis, and DNA concentration and purity were assessed using spectrophotometric methods. Qualified DNA samples were used for paired-end library construction following the manufacturer’s standard Illumina protocols and sequenced on the Illumina NovaSeq 6000 platform to generate 150-bp paired-end reads.

Raw reads were processed to remove adapter sequences, reads containing excessive ambiguous bases, and low-quality reads, thereby generating clean reads for downstream analysis. Clean reads were aligned to the *L. acutangula* reference genome using BWA-MEM v0.7.17 with default parameters, and this alignment workflow was used for SNP calling, linkage-map construction, and QTL analysis. Updated BWA-MEM2 implementations may improve computational efficiency in future resequencing analyses. The resulting SAM/BAM files were sorted and indexed using SAMtools v1.10, and PCR duplicates were marked using Picard v2.23.8 before variant calling.

Single-nucleotide polymorphisms (SNPs) were identified using GATK HaplotypeCaller v4.1.9.0 following standard best-practice procedures. The average sequencing depth was approximately 20× for each parent and 8× for each F2 individual. Only biallelic SNP loci were retained for downstream analyses. To obtain a high-confidence marker set, SNPs were filtered according to the following criteria: loci had to be polymorphic between the two parents; loci with a missing rate > 20% in the F2 population were removed; loci with read depth < 10× in the parents or < 3× in F2 individuals were excluded; genotypes with genotype quality (GQ) < 20 were discarded; and loci showing unstable or ambiguous genotype patterns were removed. The retained SNPs were formatted into a marker matrix suitable for F2 linkage analysis. Sequencing output and SNP summary statistics are provided in **Supplementary Table 1**.

### 2.4 Genetic linkage map construction

To reduce marker redundancy and improve map robustness, SNP markers showing identical genotype patterns across all retained individuals were grouped into the same bin, and one representative bin marker per bin was selected for linkage map construction. Markers with excessive missing data, unstable genotype patterns, or significant segregation distortion were excluded before grouping and ordering. Segregation distortion was tested using the chi-square test, and markers with *P* < 0.001 were removed.

The retained representative markers were assigned to linkage groups according to recombination relationships using a minimum grouping LOD threshold of 5.0. Marker ordering within each linkage group was optimized using HighMap software v1.0, and genetic distances were calculated using the Kosambi mapping function. The quality of the linkage map was evaluated based on the number of mapped markers, total map length, average inter-marker distance, maximum gap, and the continuity of marker distribution across linkage groups. Per-linkage-group summary statistics are provided in **Supplementary Table 2**, and the distribution of adjacent marker intervals is provided in **Supplementary Table 3**.

### 2.5 QTL mapping and hotspot definition

QTL mapping was performed using QTL IciMapping version 4.2 with the inclusive composite interval mapping additive and dominance model (ICIM-ADD). The walking speed was set to 1.0 cM, and the probability in stepwise regression (PIN) was set to 0.001. Genome-wide LOD thresholds were determined separately for each trait by 1,000 permutation tests at *P* = 0.05. QTLs with LOD values exceeding the corresponding trait-specific threshold were considered significant. For each significant QTL, the linkage group, peak position, LOD score, phenotypic variance explained (PVE), additive effect, dominance effect, and support interval were recorded. The confidence interval of each QTL was defined as the 1.0-LOD support interval flanking the peak.

To define major multi-trait hotspot regions, QTL confidence intervals for different traits on the same linkage group were compared, and overlapping or immediately adjacent intervals were integrated. For the hotspot on LG3, the overlapping 1.0-LOD support intervals of hotspot-associated QTLs were merged to define a consensus hotspot interval, which was subsequently used for physical anchoring and candidate gene mining.

### 2.6 Anchoring of the LG3 hotspot to the reference genome and candidate gene prioritization

The consensus hotspot interval identified on LG3 was anchored to the *L. acutangula* reference genome by integrating the genetic positions of flanking markers with their corresponding physical coordinates. Based on the physical positions of hotspot-associated markers, the LG3 hotspot was converted into a defined interval on chromosome 3.

Gene models located within the anchored physical interval were extracted from the reference genome annotation. The corresponding coding sequences and predicted protein sequences were retrieved for functional annotation. Putative gene functions were inferred by integrating multiple sources of annotation evidence, including sequence similarity searches against Swiss-Prot and NR databases, conserved domain annotation from Pfam, and functional classification from Gene Ontology (GO) and KEGG.

To incorporate positional evidence into candidate prioritization, the estimated physical position of the QTL peak was inferred by interpolation between linked genetic and physical marker positions. Genes within the hotspot interval were then classified into positional tiers according to their distance from the estimated physical peak: Tier 1, genes located within ±0.5 Mb of the estimated peak; Tier 2, genes located within ±1.0 Mb of the estimated peak; and Tier 3, the remaining genes located within the anchored hotspot interval. Candidate-gene prioritization was based on both positional proximity and biological plausibility inferred from integrated functional annotation. In addition to score- and tier-based prioritization, genes with uniquely relevant flowering-related annotations were manually retained when their functional plausibility was considered particularly strong, even if they were not among the highest-scoring peak-proximal candidates.

To assess annotation completeness, genes were considered low-information candidates when similarity-based annotation returned only generic descriptions such as “hypothetical,” “uncharacterized,” or “unknown,” or when no supporting Pfam, GO, or KEGG evidence was available. The full list of genes identified within the hotspot interval, the prioritized candidate subset, a concise summary of key flowering-related highlighted candidates, and the parental differential variants detected in the two prioritized candidate-gene intervals are provided in **Supplementary Tables 4**–**7**, respectively.

### 2.7 Regional parental sequence comparison of prioritized candidate genes

Regional sequence variation between the two parents was examined in the *Lac03g1231* and *Lac03g1281* intervals using the parental resequencing dataset and reference-based variant calls. For each gene, the analyzed interval comprised the gene body plus the 2-kb upstream region (*Lac03g1231*: chr03:16821852–16828391; *Lac03g1281*: chr03:19031801–19042112). Variants were classified according to genomic region and coding consequence based on the reference annotation, and parental differential variants were defined as sites at which the genotypes of P1–4 and P2–9 differed. Detailed results are summarized in **Supplementary Table 7**.

## 3 Results

### 3.1 Phenotypic variation and correlations among early-maturity–related traits

**Figure 1:**
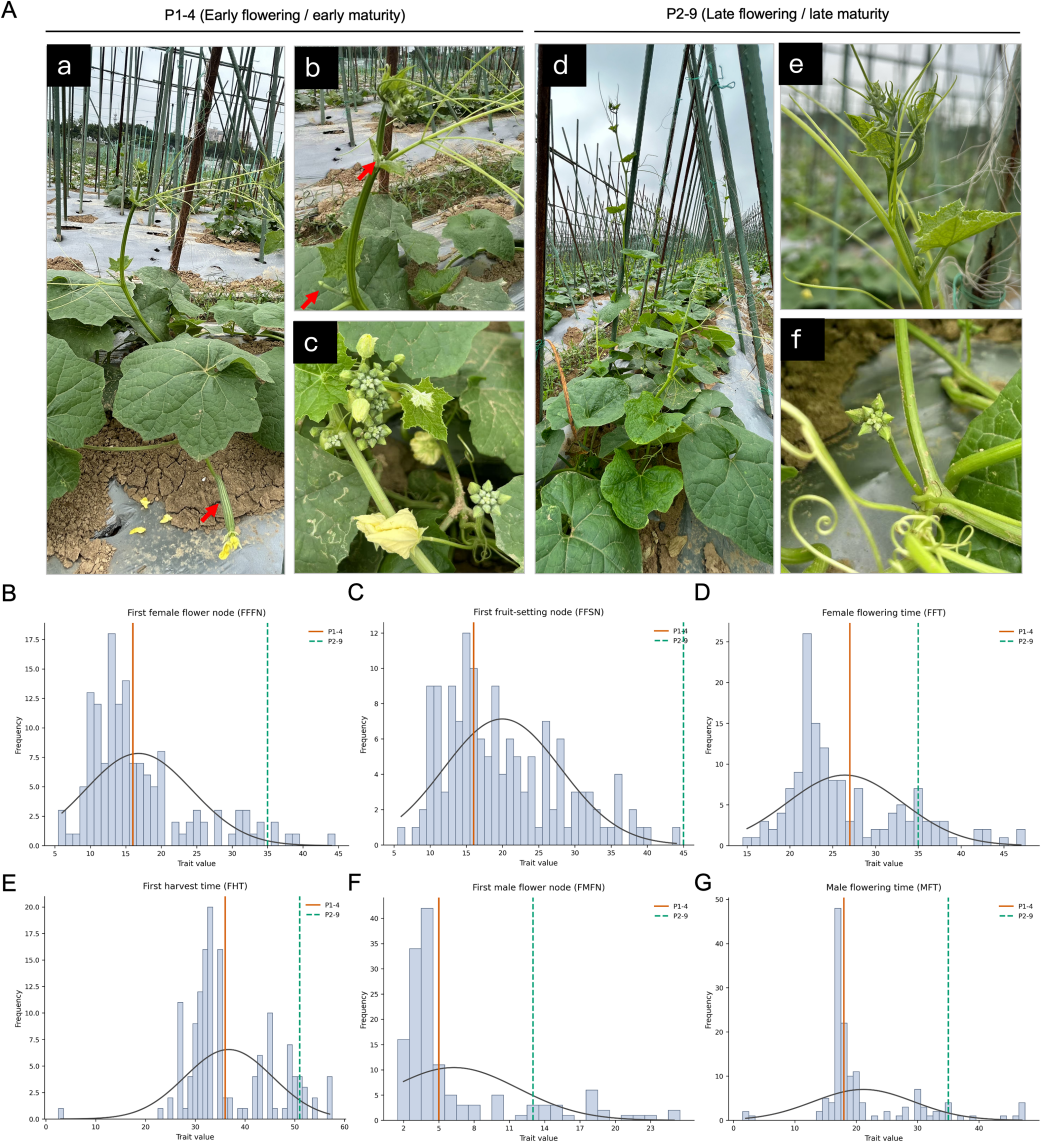
Parental phenotypes and distributions of early-maturity–related traits in the luffa F2 population. **(A)** Representative field and close-up images of the two parental lines at the reproductive transition stage. P1–4 shows early flowering and early maturity, whereas P2–9 shows late flowering and late maturity. Panels **(A–C)** show the early-maturing parent P1-4, and panels **(D–F)** show the late-maturing parent P2-9. Red arrows indicate representative early reproductive structures in P1-4. **(B–G)** Frequency distributions of six early-maturity–related traits in the F2 population, including **(B)** first female flower node (FFFN), **(C)** first fruit-setting node (FFSN), **(D)** female flowering time (FFT), **(E)** first harvest time (FHT), **(F)** first male flower node (FMFN), and **(G)** male flowering time (MFT). Histograms show the phenotypic distributions of the F2 population, and the fitted curve indicates the overall distribution pattern. Vertical solid and dashed lines indicate the parental reference values of P1–4 and P2-9, respectively, facilitating comparison between the segregating population and the parental range.

Six early-maturity–related traits were evaluated in the phenotyped F2 population of 147 plants, including first male flower node (FMFN), male flowering time (MFT), first female flower node (FFFN), female flowering time (FFT), first harvest time (FHT), and first fruit-setting node (FFSN). The two parental lines showed clear phenotypic differences for these traits, and the F2 population exhibited broad phenotypic variation between and beyond the parental values (**Supplementary Tables 8–10**). With parental reference values overlaid in **Figure 1**, the extended upper tails for FMFN, MFT, FFFN, and FFT become visually apparent, supporting the presence of transgressive segregation in these traits. All six traits showed continuous distributions, consistent with quantitative inheritance. Transgressive segregation observed for several traits indicates that both parents contributed alleles with opposite effects on different components of earliness. Trait-specific sample sizes ranged from 143 to 147 among the six phenotypes.

Frequency distributions were generally unimodal, although node-related traits (FMFN, FFFN, and FFSN) showed varying degrees of right skewness (**Figure 1**), which may reflect developmental constraints on the emergence of the first reproductive nodes. Overall, these patterns support the suitability of the population for linkage mapping of complex early-maturity–related traits.

**Figure 2:**
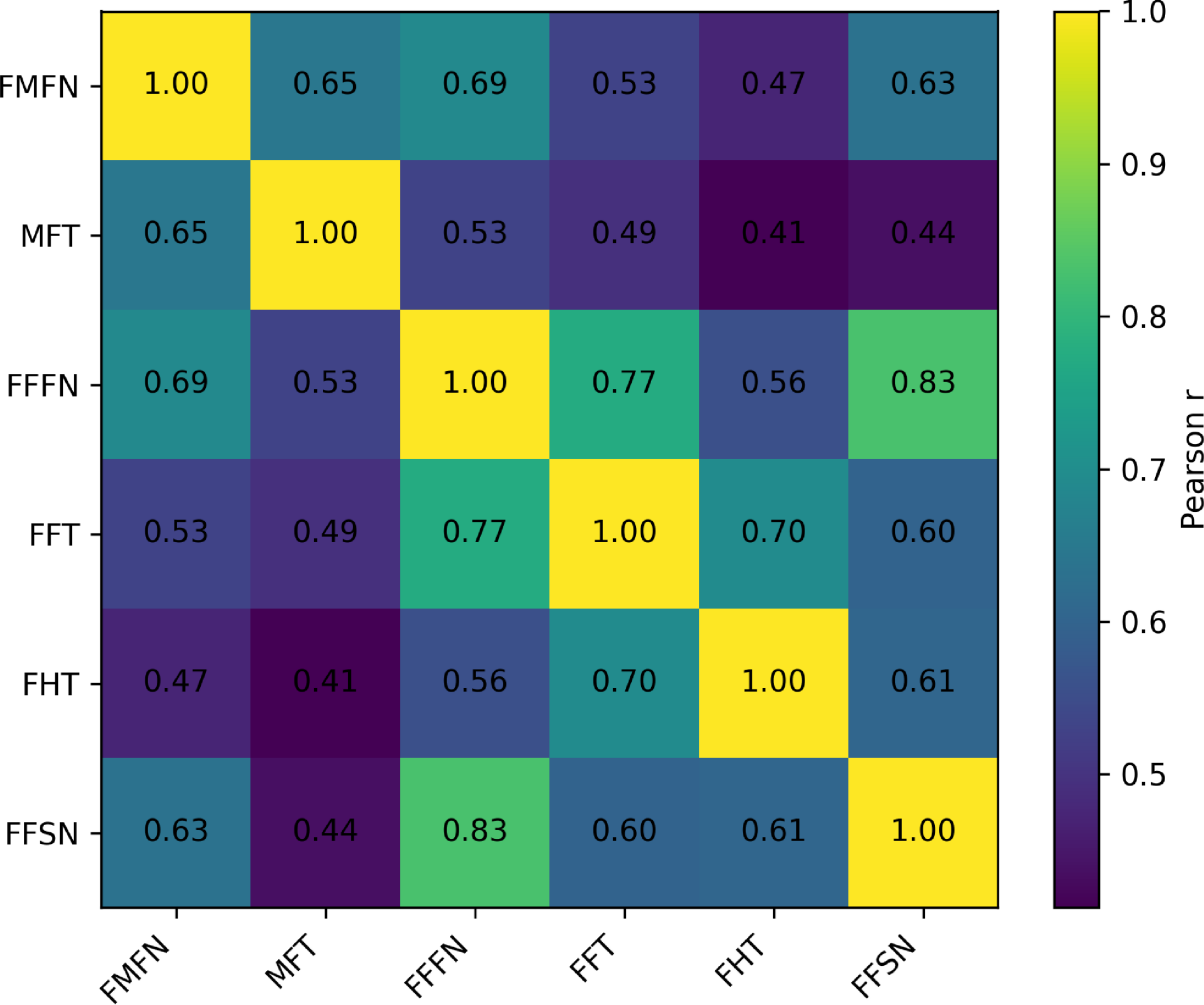
Pearson correlation matrix among early-maturity–related traits in the ridge gourd F2 population. Pairwise Pearson correlation coefficients (r) are shown as numbers within cells, and the color scale indicates correlation strength (blue to yellow). All displayed correlations were statistically significant (*P* < 0.05). Traits include first male flower node (FMFN), male flowering time (MFT), first female flower node (FFFN), female flowering time (FFT), first harvest time (FHT), and first fruit-setting node (FFSN).

Pairwise correlation analysis among traits revealed predominantly positive relationships among flowering initiation, node position, fruit setting, and harvest timing (**Figure 2**). All displayed pairwise correlations were statistically significant (all *P* < 0.05). Particularly strong associations were observed between FFFN and FFSN (r = 0.832), indicating that the node position of the first female flower was closely related to the node position of the first successful fruit set. In addition, FFT and FHT were positively correlated (r = 0.695), consistent with the developmental expectation that earlier female flowering generally contributes to earlier first harvest. These phenotypic relationships suggest that some components of early maturity may share a partially overlapping genetic basis.

### 3.2 Whole-genome resequencing, SNP discovery, and segregation-type composition of markers

Whole-genome resequencing was conducted for 146 samples, including two parents and 144 F2 individuals. In total, 692.84 Gb of clean data were generated, with high sequencing quality (mean Q30 = 94.21%) and stable GC content (mean GC = 36.95%) (**Supplementary Table 1**). Across the F2 individuals, an average of approximately 0.86 million SNPs per sample was identified, and the mean heterozygosity rate was 44.11%. These metrics indicated that the resequencing dataset was of sufficient quality for subsequent genotyping, linkage analysis, and QTL detection.

**Figure 3:**
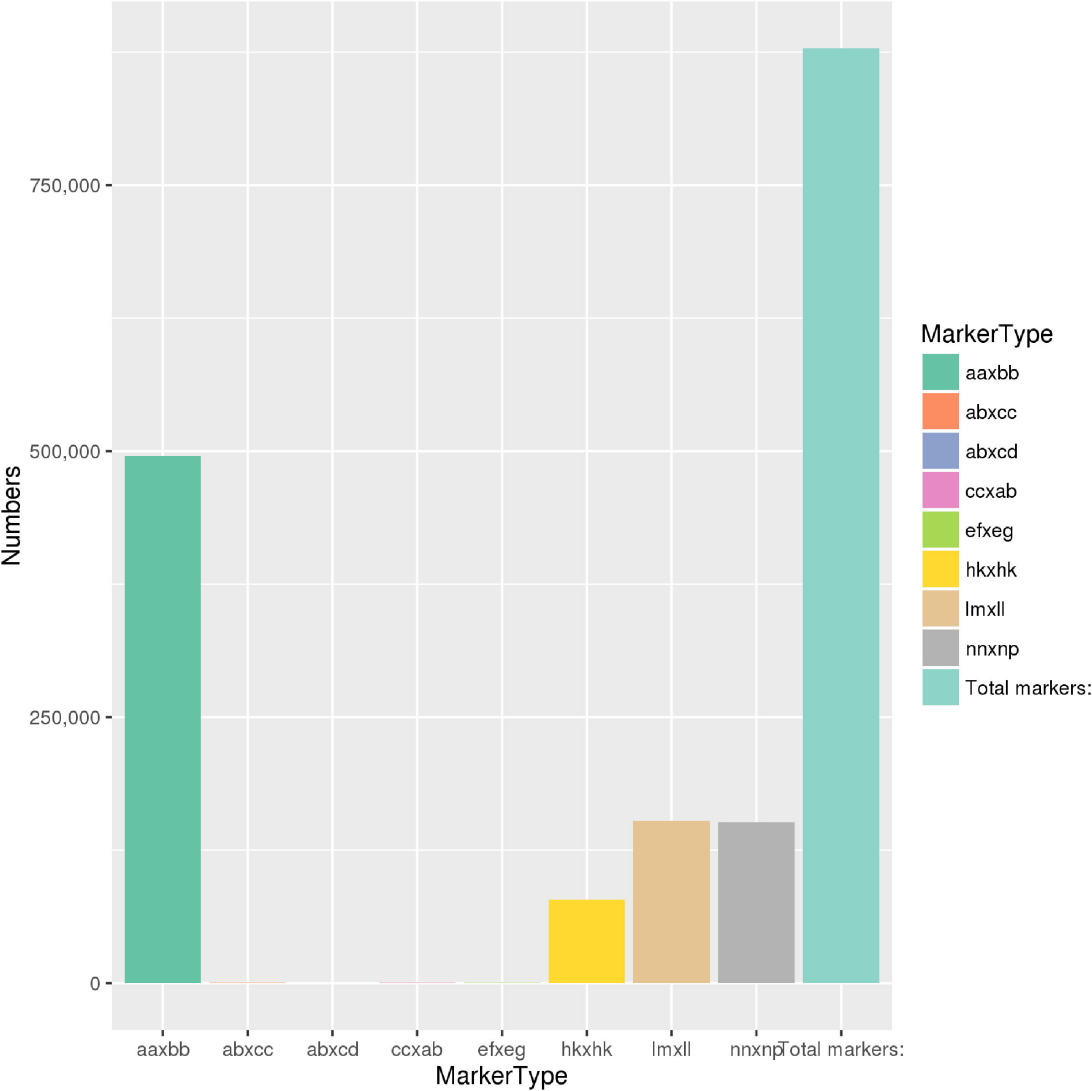
Distribution of marker segregation types used for genetic map construction. Bar plot showing the number of SNP markers classified into different segregation patterns (aa×bb, ab×cc, ab×cd, cc×ab, ef×eg, hk×hk, lm×ll, and nn×np) based on parental genotypes in the mapping population. The final bar indicates the total number of markers retained after initial classification.

A total of 878,800 polymorphic SNP markers were classified into different segregation types before linkage map construction. Among them, aa×bb markers represented the largest proportion (495,502; 56.38%), followed by lm×ll (152,724; 17.38%), nn×np (151,170; 17.20%), and hk×hk (78,585; 8.94%) (**Figure 3**). Only small numbers of markers belonged to other segregation categories, including ef×eg (398; 0.05%), ab×cc (252; 0.03%), and cc×ab (169; 0.02%), whereas ab×cd was not detected.

The predominance of aa×bb markers indicates that a large fraction of polymorphisms reflected clear allelic differentiation between the two parents. At the same time, the presence of lm×ll, nn×np, and hk×hk markers suggests that the parental lines were not completely homozygous at all loci, resulting in additional informative segregation patterns in the derived F2 population. This segregation-type composition is therefore consistent with the genetic properties of the parental materials used in this study rather than being attributable to low-quality genotyping alone.

### 3.3 High-density linkage map construction and general features of the map

After marker filtering, segregation screening, and binning of redundant loci, a total of 4,035 high-confidence representative bin markers were assigned to 13 linkage groups (LGs), consistent with the haploid chromosome number of ridge gourd (**Supplementary Table 2**). The final integrated map spanned 8,683.61 cM, with an average marker interval of 2.26 cM. Linkage group lengths ranged from 116.38 cM on LG13 to 1,508.62 cM on LG7.

Most adjacent marker intervals were relatively short, and 93.67% of marker intervals were ≤5 cM, indicating generally dense marker coverage across the linkage map (**Supplementary Table 3**). The maximum gap observed was 17.10 cM. Although marker density was adequate for genome-wide QTL scanning, the total map length was comparatively large, and this should be considered when interpreting recombination scale and QTL interval size. Nevertheless, the map provided a workable framework for subsequent QTL detection and physical anchoring analyses.

### 3.4 Linkage–genome correspondence and macro-collinearity with the reference assembly

**Figure 4:**
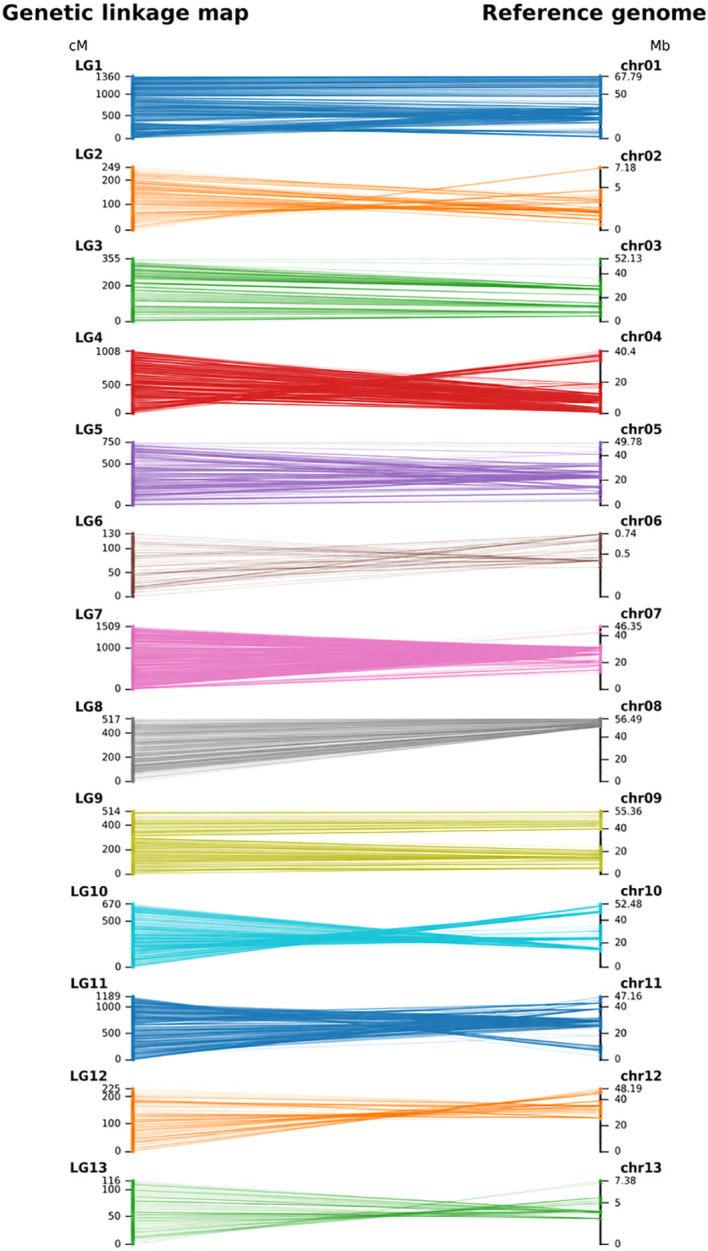
Linkage–genome correspondence between the genetic linkage map and the reference genome used in this study. Each line represents one anchored marker connecting its genetic position on the linkage map (LG1–LG13) to its physical position on the reference genome (chr01–chr13). The figure summarizes linkage group-to-chromosome correspondence directly at the marker level. Predominant one-to-one correspondence was observed between LG1–LG13 and chr01–chr13, respectively.

Mapped markers were anchored to physical coordinates and visualized in a marker-level linkage–genome correspondence plot (**Figure 4****;** **Supplementary Table 11**). Each anchored marker is represented by a connection between its genetic position on the linkage map and its physical position on the reference genome, allowing direct assessment of linkage group-to-chromosome correspondence. The anchoring pattern showed predominant one-to-one correspondence between LG1–LG13 and chr01–chr13, respectively, supporting overall agreement between the genetic map and the reference assembly.

Within these linkage group-to-chromosome correspondences, marker order generally followed an approximately monotonic relationship between genetic and physical positions, although local reversals in orientation were observed in some groups. The genetic-versus-physical position patterns of anchored markers for individual linkage groups are shown in **Supplementary Figure 1**. Such local reversals may reflect map orientation, local ordering uncertainty, or local assembly-related factors rather than broad-scale inconsistency. Overall, the correspondence analysis supports the suitability of the linkage map for genetic-to-physical projection of QTL intervals.

### 3.5 QTL mapping of early-maturity–related traits

Inclusive composite interval mapping identified eight significant QTLs for the six early-maturity–related traits (**Supplementary Tables 12, 13**). **Supplementary Table 12** summarizes the genetic peak positions, 1.0-LOD support intervals, flanking bin markers, and the projected physical positions of the flanking markers for each significant QTL. Most QTLs were located on LG3, whereas one additional locus was detected on LG8. This distribution indicates that early-maturity variation in the present population was associated primarily with one major genomic region on LG3 together with a limited number of additional background loci.

**Figure 5:**
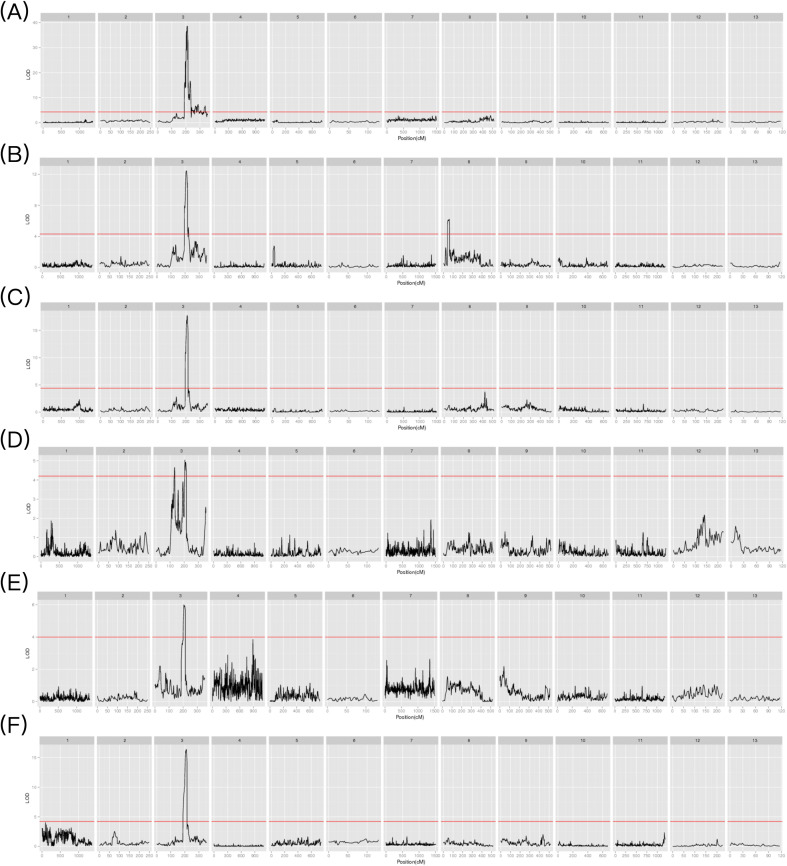
Genome-wide LOD profiles for QTL mapping of six early-maturity traits in the luffa F2 population. LOD score curves across the 13 linkage groups are shown for **(A)** first male flower node (FMFN), **(B)** male flowering time (MFT), **(C)** first female flower node (FFFN), **(D)** female flowering time (FFT), **(E)** first harvest time (FHT), and **(F)** first fruit-setting node (FFSN). The x-axis indicates genetic position (cM) within each linkage group, and the y-axis indicates the LOD score from ICIM analysis. The horizontal red line denotes the trait-specific genome-wide significance threshold determined by permutation testing; peaks exceeding the threshold indicate significant QTL positions.

A prominent region on LG3, approximately between 205 and 210 cM, was repeatedly detected for multiple traits and therefore represented the major QTL hotspot in this population. For FMFN, qFMFN3.1 peaked at 210.01 cM, with a LOD score of 38.61, a 1.0-LOD support interval of 207.4–211.9 cM, and a PVE of 57.8% (**Supplementary Table 12****;** **Figure 5**). For FFFN, qFFFN3.1 peaked at 209.01 cM (LOD = 17.71; PVE = 65.6%; 1.0-LOD interval = 208.3–212.2 cM), and for FFSN, qFFSN3.1 peaked at 210.01 cM (LOD = 16.37; PVE = 55.8%; 1.0-LOD interval = 206.0–213.2 cM). In addition, qMFT3.1 for MFT was detected at 209.01 cM (LOD = 12.44; PVE = 70.0%; 1.0-LOD interval = 204.3–214.2 cM), whereas qFFT3.1 for FFT and qFHT3.1 for FHT were detected at 205.11 cM, with support intervals overlapping the same LG3 region.

The co-occurrence of QTL peaks for multiple traits within overlapping support intervals indicates that this LG3 region contributes to several correlated components of early maturity. Because the six evaluated traits represent closely related developmental aspects of agronomic earliness, this co-localization is biologically consistent with partially shared genetic control. However, based on the present mapping resolution, it remains unclear whether the observed clustering reflects pleiotropic action of a single locus or tight linkage among multiple closely spaced loci.

Beyond the major LG3 hotspot, two additional loci were detected. The first, *qFFT3.2* for FFT, was located on LG3 at 132.41 cM (LOD = 4.65; 1.0-LOD interval = 127.4–133.9 cM), indicating that female flowering time may also be influenced by a second region on the same linkage group. The second, *qMFT8.1* for MFT, was located on LG8 at 60.71 cM (LOD = 6.22; 1.0-LOD interval = 42.4–62.6 cM) and explained a relatively small proportion of phenotypic variance (PVE = 2.8%), suggesting a minor background contribution outside the major LG3 region.

### 3.6 Projection of QTLs onto the linkage map and definition of the LG3 hotspot

**Figure 6:**
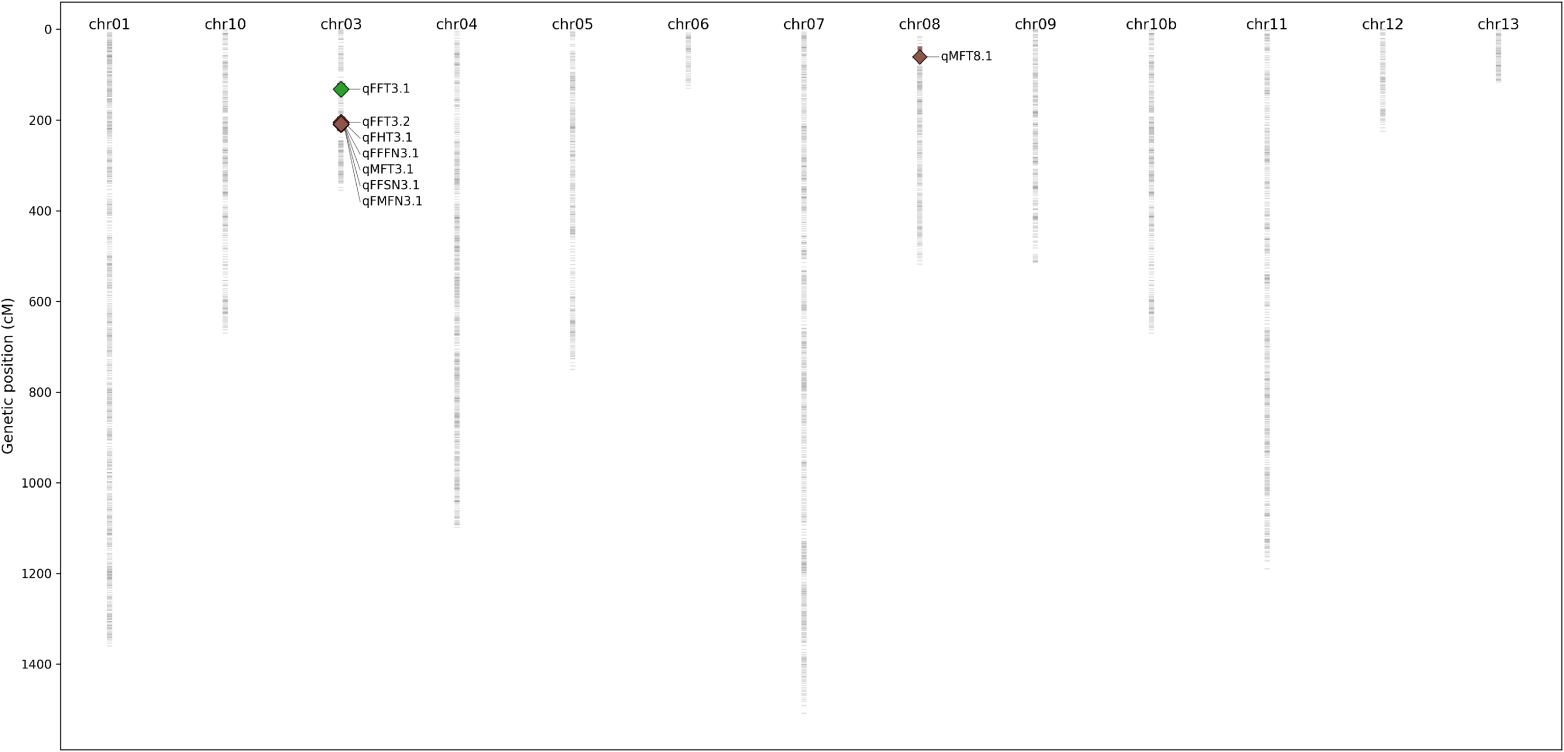
Locations of significant QTLs for early-maturity traits projected onto the luffa linkage map. The 13 linkage groups (LG1–LG13) are shown as vertical marker tracks, with genetic positions in centiMorgans (cM) on the y-axis. Significant QTLs detected by ICIM are indicated by colored diamonds at the peak positions, and the corresponding 1.0-LOD support intervals are shown as vertical segments.

To visualize the chromosomal distribution of detected QTLs, all significant QTL peaks and their 1.0-LOD support intervals were projected onto the linkage map (**Figure 6**). This integrated presentation showed that most trait-associated intervals clustered on LG3, highlighting the multi-trait nature of the major region detected in this study. Several QTLs shared overlapping intervals in the approximately 205–210 cM region, consistent with repeated detection of the same chromosomal segment across flowering-, node-, and harvest-related traits.

To define a unified region for downstream physical anchoring, the 1.0-LOD support intervals of hotspot-associated QTLs on LG3 were merged. The resulting consensus hotspot spanned 201.3–215.4 cM (**Supplementary Table 14**). This interval was supported by multiple traits, including the narrower intervals of qFMFN3.1, qFFFN3.1, and qFFSN3.1, together with the broader interval of qFHT3.1. The merged hotspot interval was subsequently used for physical projection and candidate gene mining.

### 3.7 Physical anchoring of the LG3 hotspot and candidate gene prioritization

The merged LG3 hotspot interval was projected onto the reference genome and corresponded to Chr03: 15,820,849–22,850,576 bp, spanning approximately 7.03 Mb. According to the genome annotation, 136 annotated genes were located within this interval (**Supplementary Table 4**). Using the estimated physical peak position (~20.01 Mb) as a reference, genes within the hotspot were classified into positional tiers: 6 genes in Tier 1 (within ±0.5 Mb of the estimated peak), 16 genes in Tier 2 (within ±1.0 Mb, including Tier 1), and the remaining 114 genes in Tier 3.

Integrated annotation using Swiss-Prot, NR, Pfam, GO, and KEGG showed that annotation depth varied among genes in the interval. Under a stringent criterion, 18 genes were classified as low-information candidates because they had only generic or uninformative annotations and lacked supporting domain or pathway evidence; among these, 7 genes lacked annotation support across all merged evidence fields (**Supplementary Table 4**). This result indicates that positional proximity alone is insufficient for candidate prioritization and that functional annotation depth remains uneven across the hotspot interval.

Based on the integrated positional and functional information, two genes were retained as biologically plausible candidates for further study. *Lac03g1281* (*Lac03g1281.1*), annotated as an Agamous-like MADS-box protein AGL80, was located within the peak-proximal Tier 2 interval and therefore represented a candidate supported by both positional and functional evidence. In addition, *Lac03g1231* (*Lac03g1231.1*) showed annotation evidence consistent with an FT-like florigen and PEBP-domain protein. Although *Lac03g1231* was located outside the ±1 Mb core interval and was therefore assigned to Tier 3, it was manually retained because its annotation was uniquely consistent with an FT/PEBP flowering integrator, making it biologically noteworthy within the broader hotspot interval. Its putative flowering-related function therefore supported its retention as a candidate of biological interest despite its lower positional priority. Nevertheless, this prioritization was based on positional and annotation evidence only and should not be interpreted as direct proof of causality.

Regional parental sequence comparison further showed that both candidate intervals were polymorphic between P1–4 and P2-9 (**Supplementary Table 7**). In the *Lac03g1231* interval (chr03:16821852–16828391), 40 parental differential SNPs were detected, including 21 upstream variants, 18 intronic variants, and one synonymous exonic variant. In the *Lac03g1281* interval (chr03:19031801–19042112), 16 parental differential SNPs were detected, including three upstream variants, 12 intronic variants, and one synonymous exonic variant. No parental differential nonsynonymous, splice-site, or start/stop-codon variants were detected in either prioritized candidate gene.

Together, these results define a prioritized candidate framework but do not establish causality. The parental comparison results provide supportive regional allelic evidence, yet they do not identify an obvious coding-sequence causal variant in either prioritized gene. Developmental-stage expression analysis, finer-resolution mapping, and direct functional validation remain necessary to determine whether either gene underlies the LG3 hotspot.

## 4 Discussion

### 4.1 A major LG3 hotspot underlies multiple early-maturity–related traits in ridge gourd

Multiple QTLs associated with early-maturity–related traits were detected within an overlapping interval on LG3, and integration of their 1.0-LOD support intervals defined a major hotspot spanning 201.3–215.4 cM. After projection onto the reference genome, this region corresponded to a physical interval of approximately 7.03 Mb on chromosome 3. The repeated detection of QTLs for FMFN, FFFN, FFSN, MFT, FFT, and FHT within this region suggests that an important component of earliness variation in the analyzed population is concentrated within a relatively restricted genomic segment.

The co-localization of QTLs for multiple correlated traits is biologically plausible because flowering initiation, reproductive node position, fruit setting, and first harvest timing are developmentally connected processes. In QTL studies of complex traits, such clustering can arise from pleiotropic effects of a single underlying locus or from tight linkage among multiple loci controlling related developmental processes (Schulthess et al., 2017). Based on the current mapping resolution, these two possibilities cannot yet be distinguished in ridge gourd. Nevertheless, the recurrent detection of this region across several traits indicates that LG3 is a key genomic target for understanding and manipulating agronomic earliness in this crop.

The relatively large phenotypic variance explained by several LG3 QTLs should be interpreted cautiously. In finite mapping populations, effect sizes estimated from the same dataset used for QTL detection may be inflated, a phenomenon commonly referred to as the Beavis effect or upward sampling bias (Xu, 2003; Utz et al., 2000; Melchinger et al., 2004). This consideration is particularly important because the mapping population size was moderate and phenotyping was conducted in a single field environment at one location, without multi-year or multi-location evaluation. Although the LG3 hotspot is strongly supported as a major region in this population, its precise effect size and its stability across years and locations remain to be validated.

### 4.2 The LG3 hotspot in the context of reproductive development in cucurbit crops

In cucurbit crops, traits related to earliness often reflect coordinated transitions in flowering initiation, sex expression, node differentiation, fruit setting, and early reproductive development. Previous genetic and molecular studies in cucumber and melon have shown that these processes are regulated by interconnected developmental pathways rather than by completely independent modules. In particular, ethylene biosynthesis and signaling play central roles in female flower development and sex expression, with ACC synthase (ACS) and ACC oxidase (ACO) genes acting as major regulatory components (Trebitsh et al., 1997; Boualem et al., 2008, 2015; Chen et al., 2016; Pan et al., 2021). These studies support the expectation that loci affecting female flowering and fruit-setting traits can have substantial downstream effects on overall earliness.

In addition to ethylene-related pathways, flowering time and reproductive architecture in cucurbits are also influenced by conserved flowering integrators and meristem determinacy regulators. FT/PEBP family genes act as central components of floral induction pathways, while TFL1-like genes influence determinacy, shoot architecture, and the transition between vegetative and reproductive development (Kobayashi et al., 1999; Kardailsky et al., 1999; Corbesier et al., 2007; Wigge, 2011; Wen et al., 2019). Accordingly, a multi-trait hotspot in ridge gourd is consistent with the idea that early-maturity–related traits share partially overlapping developmental control systems, as has also been observed in other cucurbits.

The LG3 region is supported by QTL co-localization rather than by direct mechanistic evidence for any specific pathway. Although the pattern observed on LG3 is consistent with coordinated control of several reproductive traits, current evidence does not allow discrimination between a single pleiotropic regulator, a cluster of tightly linked genes, or a combination of both. Interpretation of the hotspot should therefore remain anchored in positional evidence and broad biological plausibility rather than strong functional inference.

### 4.3 Candidate gene landscape of the LG3 interval and the limits of annotation-based prioritization

Anchoring the consensus LG3 hotspot to the reference genome used in this study yielded a physical interval of approximately 7.03 Mb containing 136 annotated genes. This gene number is typical of a first-pass QTL interval of this size and immediately illustrates a practical challenge in moving from QTL detection to causal-gene identification. Even after integrating multiple annotation sources, including Swiss-Prot, NR, Pfam, GO, and KEGG, the functional interpretability of genes across the interval remained uneven.

Using a stringent definition based on low-information similarity annotations and the absence of supporting domain or pathway evidence, 18 genes were classified as unclear or low-information candidates, including 7 genes lacking annotation support across all merged evidence fields. Importantly, such annotation uncertainty was not limited to distal portions of the interval but also occurred among genes located close to the estimated physical peak. This finding indicates that positional proximity alone is insufficient for candidate prioritization and that annotation incompleteness remains an important limitation in ridge gourd, as in many non-model or less extensively curated crop genomes.

The tiered prioritization framework therefore serves as a practical filter rather than a definitive ranking of causality. It combines positional evidence with functional plausibility to reduce the candidate search space. Additional evidence, particularly parental allelic variation, expression profiling at relevant developmental stages, and higher-resolution recombination mapping, will be required to refine the candidate list further. Regional parental sequence comparison of *Lac03g1231* and *Lac03g1281* detected multiple differential variants in both intervals, but the exonic differences were synonymous substitutions only, and no parental differential nonsynonymous, splice-site, or start/stop-codon variants were identified (**Supplementary Table 7**). These results strengthen the candidate framework with supportive allelic evidence but remain insufficient for causal inference.

### 4.4 Two biologically plausible flowering-related candidates within the hotspot

Among the genes currently annotated within the LG3 interval, two candidates were considered particularly noteworthy based on the combined positional and functional evidence. The first, *Lac03g1231*, showed annotation evidence consistent with an FT-like florigen and PEBP-domain-related protein. Although *Lac03g1231* was not among the highest-scoring peak-proximal candidates, it was retained because its annotation was uniquely consistent with an FT/PEBP flowering integrator, which is highly relevant to flowering transition. FT-like genes are well-established regulators of floral induction and long-distance flowering signals in angiosperms (Kobayashi et al., 1999; Kardailsky et al., 1999; Corbesier et al., 2007; Wigge, 2011). Because floral transition is a central component of agronomic earliness, an FT-like homolog within the hotspot is a biologically credible candidate. In cucurbits, FT/TFL1-related modules have also been implicated in the regulation of flowering transition and growth habit, further supporting the relevance of this gene class for the traits evaluated here (Wen et al., 2019).

The second candidate, *Lac03g1281*, was annotated as an AGL80-like MADS-box transcription factor. MADS-box genes are widely involved in floral organ development, reproductive phase progression, and developmental identity programs. Although AGL80 in Arabidopsis has been studied more in relation to female gametophyte and seed development than in canonical flowering-time control, its membership in a reproductive-development-associated transcription factor family makes Lac03g1281 a plausible candidate within the ridge gourd hotspot (Portereiko et al., 2006). In addition, *Lac03g1281* is positioned within the peak-proximal Tier 2 interval, which strengthens its priority from a positional standpoint.

Both genes should therefore be regarded as prioritized candidates rather than validated causal genes. *Lac03g1231* is functionally attractive but lies outside the ±1 Mb core window, whereas Lac03g1281 is peak-proximal but its specific relevance to ridge gourd earliness remains inferential. Regional parental sequence comparison confirmed that both candidate intervals are polymorphic between P1–4 and P2-9, but the exonic parental differences detected in the two prioritized genes were synonymous substitutions only (**Supplementary Table 7**). These data support continued evaluation of both genes but do not yet demonstrate that either gene underlies the LG3 QTL hotspot.

### 4.5 Breeding value of the LG3 hotspot and prospects for marker development

From an applied perspective, the identification of a major QTL hotspot associated with multiple early-maturity–related traits has potential value for ridge gourd breeding. Because the hotspot influences traits that are agronomically integrated—flowering node position, flowering time, fruit setting, and time to first harvest—markers closely linked to this region may enable more efficient selection for earliness-related performance than selection based on any single trait alone. This is particularly useful in field breeding, where earliness is often evaluated as a composite phenotype influenced by environmental conditions.

A practical next step is the development of breeder-friendly markers from informative polymorphisms within the hotspot interval. Kompetitive allele-specific PCR (KASP) markers are especially suitable for this purpose because they are flexible, cost-effective, and widely used for crop genotyping and marker-assisted selection (Semagn et al., 2014; He et al., 2014). Recent cucurbit fine-mapping studies further show that physically anchored major QTL intervals can be narrowed efficiently and converted into reliable breeder-oriented markers when dense linkage mapping is combined with regional polymorphism analysis and recombinant screening (Lv et al., 2025). Once validated in segregating and breeding populations, tightly linked KASP markers could support early-generation screening and accelerate the introgression of favorable alleles associated with earlier reproductive transition and earlier harvest.

At present, however, the relatively broad interval still limits immediate deployment of a small, highly reliable diagnostic marker set. Additional interval reduction and validation will therefore be required before routine breeding application. Even so, the hotspot identified here provides a clear starting point for marker development and establishes a useful genomic target for future ridge gourd improvement.

## 5 Conclusions

A high-density genetic linkage map of ridge gourd was constructed from an F2 population and whole-genome resequencing, and eight QTLs for six early-maturity–related traits were identified. A major multi-trait hotspot on LG3 was repeatedly detected, indicating that this region plays an important role in early-maturity variation in the studied population. The hotspot was anchored to a 7.03 Mb interval on chromosome 3 containing 136 annotated genes, from which Lac03g1231 and Lac03g1281 were prioritized as biologically plausible candidates. Regional parental sequence comparison showed that both candidate intervals harbor parental polymorphisms, but no parental differential nonsynonymous, splice-site, or start/stop-codon variants were detected in the two prioritized genes. These results provide a genetic basis for understanding early maturity in ridge gourd and offer a starting point for future fine mapping, developmental-stage expression analysis, functional validation, and marker-assisted breeding.

## Data Availability

The raw whole-genome resequencing reads generated in this study have been deposited in the NCBI Sequence Read Archive (SRA) under BioProject accession number PRJNA1469353 and SRA accession numbers SRR38795095-SRR38795240.

## Supplementary material

Supplementary Figure 1 Within-linkage-group relationships between genetic position and physical position of anchored markers. Faceted scatter plots show the genetic positions (cM) and physical positions (Mb) of anchored markers for individual linkage groups. Panel titles indicate the primary corresponding chromosome and the number of anchored markers. This supplementary figure complements **Figure 4** by showing local marker-order relationships within individual linkage groups.
