# Supplementary figures and images for: A high-density SNP linkage map reveals a major LG3 QTL hotspot controlling early-maturity–related traits in ridge gourd (Luffa acutangula)
Source: Front Plant Sci. 2026 May 28;17:1832490. doi: 10.3389/fpls.2026.1832490 (PMC13254177; doi:10.3389/fpls.2026.1832490)

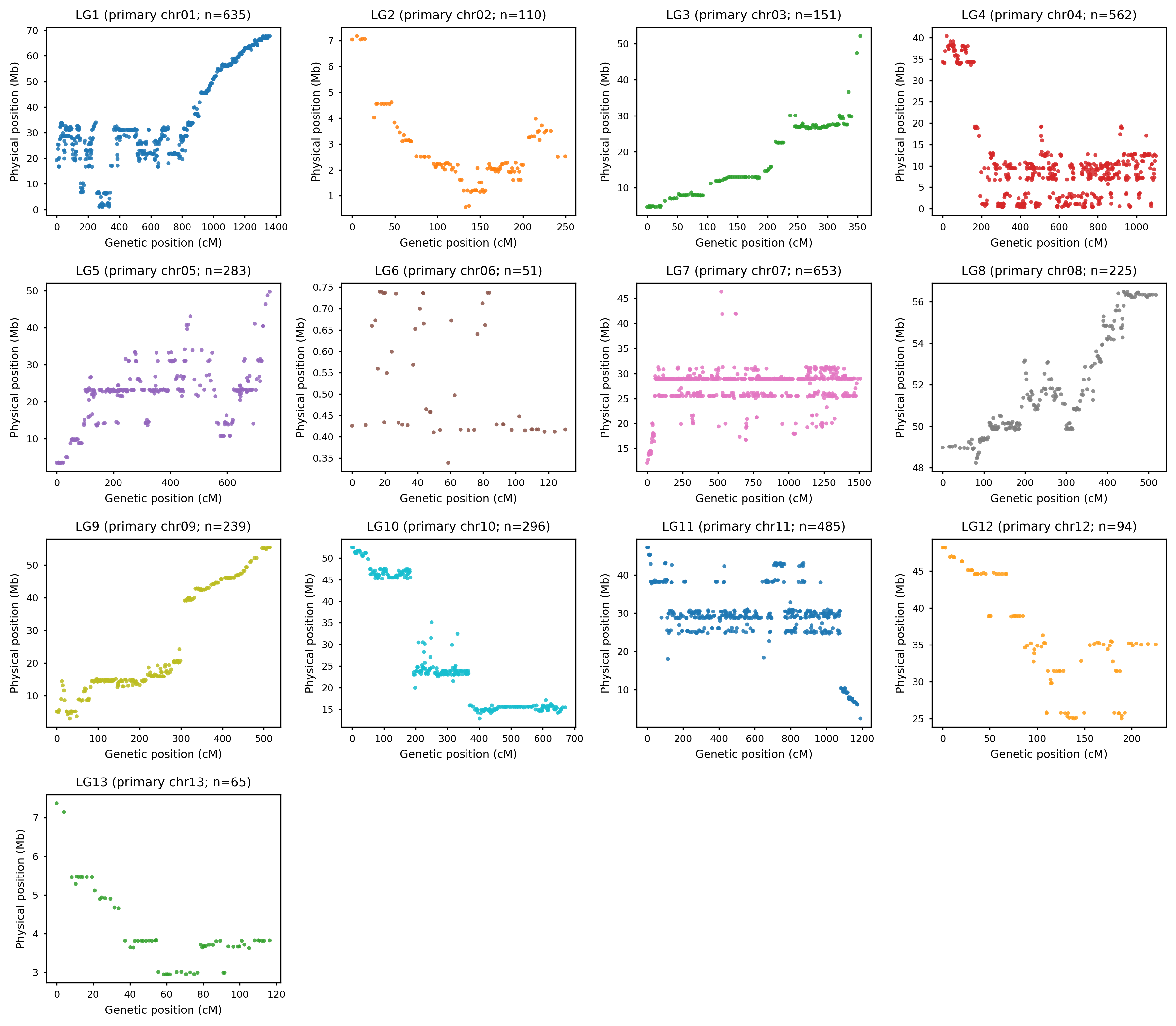

Supplement: Supplementary Figure 1 — Within-linkage-group relationships between genetic position and physical position of anchored markers. Faceted scatter plots show the genetic positions (cM) and physical positions (Mb) of anchored markers for individual linkage groups. Panel titles indicate the primary corresponding chromosome and the number of anchored markers. This supplementary figure complements Figure 4 by showing local marker-order relationships within individual linkage groups. [file Image1.tif]
